# Supplementary material for: Haberlea rhodopensis Extract Tunes the Cellular Response to Stress by Modulating DNA Damage, Redox Components, and Gene Expression
Source: Int J Mol Sci. 2023 Nov 4;24(21):15964. doi: 10.3390/ijms242115964 (PMC10647427; doi:10.3390/ijms242115964)
Supplement: Supplementary file 1 [file ijms-24-15964-s001.zip › ijms-2639953-supplementary.pdf]

Haberlea rhodopensis Extract Tunes the Cellular Response to Stress by Modulating DNA Damage, Redox Components, and Gene Expression

Dessislava Staneva [1#], Neli Dimitrova [2#], Borislav Popov [2], Albena Alexandrova [3], Milena Georgieva [1], and George Miloshev [1*]

Supplementary Materials

Table S1. Gradient final conditions in myconaside determination using HPLC

Time (min)	Flow (mL/min)	%A	%B
0	0.6	80.0	20.0
12	0.6	80.0	20.0
15	0.6	50.0	50.0
17	0.6	80.0	20.0
20	0.6	80.0	20.0

(A)

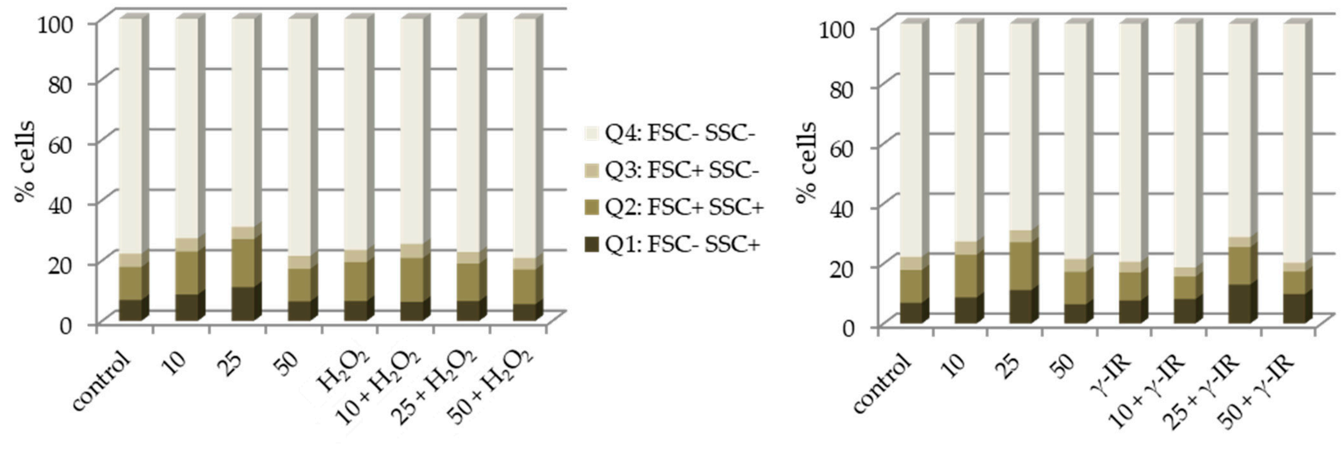

(B)

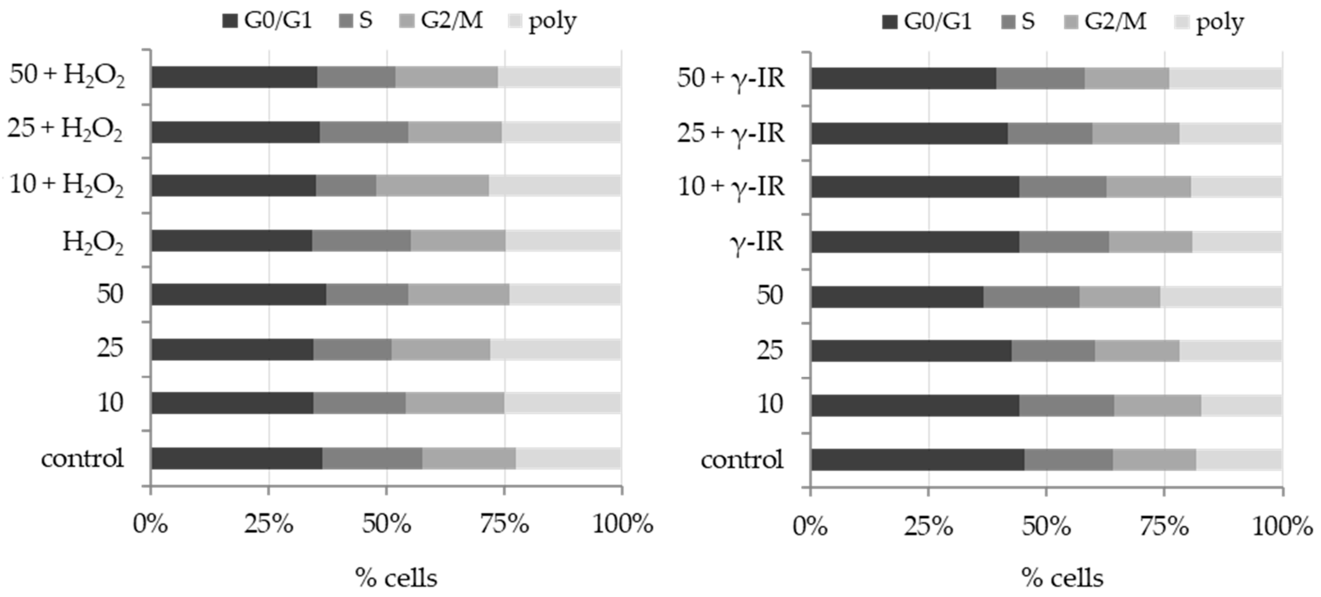

Figure S1. Cell morphology and cell cycle analyses by Flow cytometry. HeLa cells were acquired after treatment with HRE (10, 25, and 50 μg/ml) and the stressor separately or in combinations. **(A)** Distribution of cell populations according to the cellular size and granularity (FSC-H and SSC-H). **(B)** Distribution of cell populations in the cell cycle phases based on the DNA content.
